# Einsatz der kontrastmittelverstärkten Mammographie in der Brustkrebsdiagnostik

**DOI:** 10.1007/s00117-023-01222-8

**Published:** 2023-10-27

**Authors:** Uwe Fischer, Felix Diekmann, Thomas Helbich, Heike Preibsch, Michael Püsken, Evelyn Wenkel, Susanne Wienbeck, Eva Maria Fallenberg

**Affiliations:** 1 Diagnostisches Brustzentrum Göttingen, Göttingen, Deutschland; 2 Institut für Radiologische Diagnostik, Krankenhaus St. Joseph-Stift, Schwachhauser Heerstr. 54, 28209 Bremen, Deutschland; 3 Universitätsklinik für Radiologie und Nuklearmedizin, Abteilung für Allgemeine und Pädiatrische Radiologie, Medizinische Universität Wien/AKH WIEN, Währinger Gürtel 18–20, 1090 Wien, Österreich; 4 Diagnostische und Interventionelle Radiologie, Universitätsklinikum Tübingen, Hoppe-Seyler-Str. 3, 72076 Tübingen, Deutschland; 5 Institut für Diagnostische und Interventionelle Radiologie, Uniklinik Köln, Kerpener Str. 62, 50937 Köln, Deutschland; 6 Medizinische Fakultät, Friedrich-Alexander-Universität Erlangen-Nürnberg (FAU), Erlangen, Deutschland; 7 Radiologie München, München, Deutschland; 8 Radiologie Schwarzer Bär MVZ, Schwarzer Bär 8, 30449 Hannover, Deutschland; 9 Institut für Diagnostische und Interventionelle Radiologie, Medizinische Hochschule Hannover, Carl-Neuberg-Str. 1, 30625 Hannover, Deutschland; 10 Institut für diagnostische und interventionelle Radiologie, School of Medicine & Klinikum rechts der Isar Technische Universität München (TUM), Ismaninger Str. 22, 81675 München, Deutschland

**Keywords:** Mammakarzinom, Früherkennung, Radiologische Bildgebung, Iodhaltige Kontrastmittel, Patientenaufklärung, Breast cancer, Cancer screening, Radiological imaging, Iodinated contrast media, Patient education

## Abstract

**Hintergrund:**

Die kontrastmittelverstärkte Mammographie (CEM) ist ein Untersuchungsverfahren, das nach peripher-venöser Applikation eines iodhaltigen Kontrastmittels (iKM) die verbesserte Darstellung intramammärer Tumoren ermöglicht.

**Fragestellung und Methode:**

Die Autor:innen diskutieren den aktuellen Stellenwert der CEM.

**Ergebnisse:**

In Studien konnten für die CEM Vorteile bei der Brustkrebsdiagnostik gegenüber der Mammographie insbesondere bei Frauen mit sehr dichtem Drüsengewebe gezeigt werden. Das Indikationsspektrum zur CEM kann gegenwärtig in Abhängigkeit davon gesehen werden, ob eine qualitätsgesicherte Magnetresonanztomographie (MRT) der Mamma zur Verfügung steht oder nicht. Mit Option auf eine qualitätsgesicherte Mamma-MRT reduzieren sich die Indikationen für eine CEM auf Konstellationen, in denen die MRT nicht einsetzbar ist. Für das Brustkrebs-Screening wird der Einsatz der CEM gegenwärtig kritisch gesehen. Dies kann sich ändern, wenn in Kürze Ergebnisse und aktualisierte Bewertungen umfangreicher CEM-Studien in Europa und USA vorliegen. Patientinnen sind über die iKM-Gabe aufzuklären. Da die iKM-Gabe bei der CEM in ähnlicher Art und Weise erfolgt wie bei anderen etablierten Bildgebungsverfahren, ist aus Sicht der Autoren davon auszugehen, dass die Verwendung der iKM für die CEM unter Berücksichtigung der allgemeinen Kontraindikationen unproblematisch ist.

**Schlussfolgerung:**

Künftig könnte der CEM in der Brustkrebsdiagnostik eine größere Bedeutung zukommen, da dieses Verfahren diagnostische Vorteile gegenüber der konventionellen Mammographie aufweist. Als Vorteil der CEM gilt die hohe Verfügbarkeit. Für MR-Nutzer füllt die CEM schon jetzt bestehende Lücken bei Vorliegen von Kontraindikationen oder Nichtdurchführbarkeit der MRT aus anderen Gründen.

## Abstract (en)

**Background:**

Contrast-enhanced mammography (CEM) is an imaging method that is able to improve visualization of intramammary tumors after peripheral venous administration of an iodine-containing contrast medium (ICM).

**Objectives and methods:**

The current significance of CEM is discussed.

**Results:**

Studies were able to show an advantage of CEM in the diagnosis of breast cancer compared to mammography, especially for women with dense breasts. Indications for CEM currently depend on the availability of magnetic resonance imaging (MRI). If MRI is available, CEM is indicated in those cases when MRI cannot be performed. Use of CEM for breast cancer screening is currently viewed critically. This view can change when results and updated assessments of large CEM studies in Europe and USA become available. Patients must be informed about the use of an ICM. As ICM administration for CEM is carried out in a similar manner to established imaging methods, the authors expect the use of ICM for CEM to be unproblematic as long as general contraindications are adhered to.

**Conclusions:**

In the future, CEM could have greater importance for the diagnosis of breast cancer, as this imaging method has diagnostic advantages compared to conventional mammography. A great advantage of CEM is its availability. For those who use breast MRI, CEM is helpful when MRI is not feasible due to contraindications or other reasons.

In der Brustkrebsdiagnostik ermöglichen kontrastmittelverstärkte Bildgebungsverfahren bei Frauen mit dichten Gewebestrukturen eine bessere Darstellung intramammärer Tumoren im Vergleich zu kontrastmittelfreien Verfahren wie der Mammographie. Neben der kontrastmittelverstärkten Magnetresonanztomographie (MRT) könnte hier die kontrastmittelverstärkte Mammographie (CEM) zukünftig an Bedeutung gewinnen. Für deren klinische Implementierung spielen die technischen Voraussetzungen genauso eine Rolle wie geeignete Indikationen, Dosis und Befundung, die im Folgenden näher beleuchtet werden.

Mit der CEM steht ein Untersuchungsverfahren zur Verfügung, das nach peripher-venöser Applikation eines iodhaltigen Kontrastmittels (iKM) die verbesserte Darstellung intramammärer Tumoren ermöglicht. Die CEM nutzt die iKM-Aufnahme von Tumoren zusätzlich zu den rein morphologischen Kriterien, um Tumoren nachzuweisen. Die CEM zeigt sich – wie auch andere Kontrastmittel(KM)-verstärkte Methoden – der konventionellen Mammographie insbesondere bei sehr dichten Drüsengewebsstrukturen deutlich überlegen. Die European Society of Breast Imaging (EUSOBI) empfiehlt, Frauen mit sehr dichten Drüsengewebsstrukturen über die limitierte Aussagekraft der Mammographie zu informieren und ein Verfahren wie die KM-verstärkte Magnetresonanztomographie (KM-MRT) einzusetzen [20]. In Deutschland dürfte die Aussagekraft der Mammographie bei mindestens 2 Mio. screeningberechtigten Frauen eingeschränkt sein. Hier könnte auch der CEM eine Bedeutung zukommen, da dieses Verfahren z. B. hinsichtlich der Verfügbarkeit leichter ausgebaut werden kann [14, 15]. Aus diesem Grund wird versucht, den aktuellen Stellenwert dieses Verfahrens mit Blick auf die Angaben in der Literatur, aber auch vor dem Hintergrund eigener Erfahrungen zu bestimmen.

## Verfügbarkeit

Neben einem CEM-fähigen Mammographie-Gerät wird geschultes Personal für die Kontrastmittelinjektion und die Durchführung der CEM benötigt. Den Autor:innen sind, Stand Juli 2022, in Deutschland mindestens 25 im Einsatz befindliche CEM-Geräte bekannt. Für Österreich wird derzeit von mindestens 14 CEM-Geräten ausgegangen, davon 7 an Instituten (ca. 6 % aller Mammographie-Geräte), die am österreichischem Brustkrebs-Früherkennungsprogramm teilnehmen. Deutlich mehr CEM-Geräte dürften in den USA vorhanden sein. Hier lag die Schätzung für 2019 bereits bei mehr als 100 Geräten.

## Aufklärung der Patientinnen

Patientinnen sollten über potenzielle unerwünschte iKM-Nebenwirkungen aufgeklärt werden. Diese bedingen die konsekutiv notwendige ärztliche Erreichbarkeit während der Untersuchung. In den vergangenen Jahren waren sämtliche iKM für die CEM nicht zugelassen, sodass hierüber formal informiert werden musste. Iopromid (Ultravist®-300, -370, Bayer AG, Leverkusen, Deutschland) erhielt am 23.01.2023 als erstes iKM die Zulassungserweiterung für CEM in der EU; in Deutschland ist Ultravist® seit Februar 2023 für die CEM zugelassen [4]. Weitere nationale Zulassungen innerhalb der EU werden nun zeitnah erwartet.

Auf die KM-MRT als alternatives diagnostisches Untersuchungsverfahren mit besseren Ergebnissen bei fehlender Verwendung ionisierender Strahlung muss im Einklang mit rechtlichen Vorgaben ebenfalls hingewiesen werden. In Deutschland ist dies im Bürgerlichen Gesetzbuch (BGB), § 630e Aufklärungspflichten, Punkt (1) entsprechend geregelt.

## Maßnahmen vor Einsatz der CEM

Es ist davon auszugehen, dass die Verwendung von iKM unter Berücksichtigung der allgemeinen Kontraindikationen auch für die CEM unproblematisch ist, da diese für den Einsatz bei anderen röntgenbasierten und computertomographischen Untersuchungsverfahren zugelassen sind und die iKM-Gabe bei der CEM in ähnlicher Art und Weise erfolgt. Hierzu passen die neuesten Leitlinien der European Society of Urogenital Radiology (ESUR) für den KM-Einsatz. Sie sehen die Bestimmung des Thyreoidea-stimulierenden Hormons (TSH) und von Kreatinin vor der iKM-Gabe nicht mehr zwingend vor [24]. Allerdings sollten Patientinnen mit dem klinischen Risiko einer Schilddrüsenüberfunktion eine TSH-Bestimmung erhalten, und vor allem bei schwerkranken Patientinnen mit Herz- oder Niereninsuffizienz sollte das Kreatinin bestimmt werden. Auch bei Metformin-Einnahme ist der Einsatz von iKM gemäß ESUR-Guidelines möglich [6].

## Prinzip der CEM

**Figure 1:**
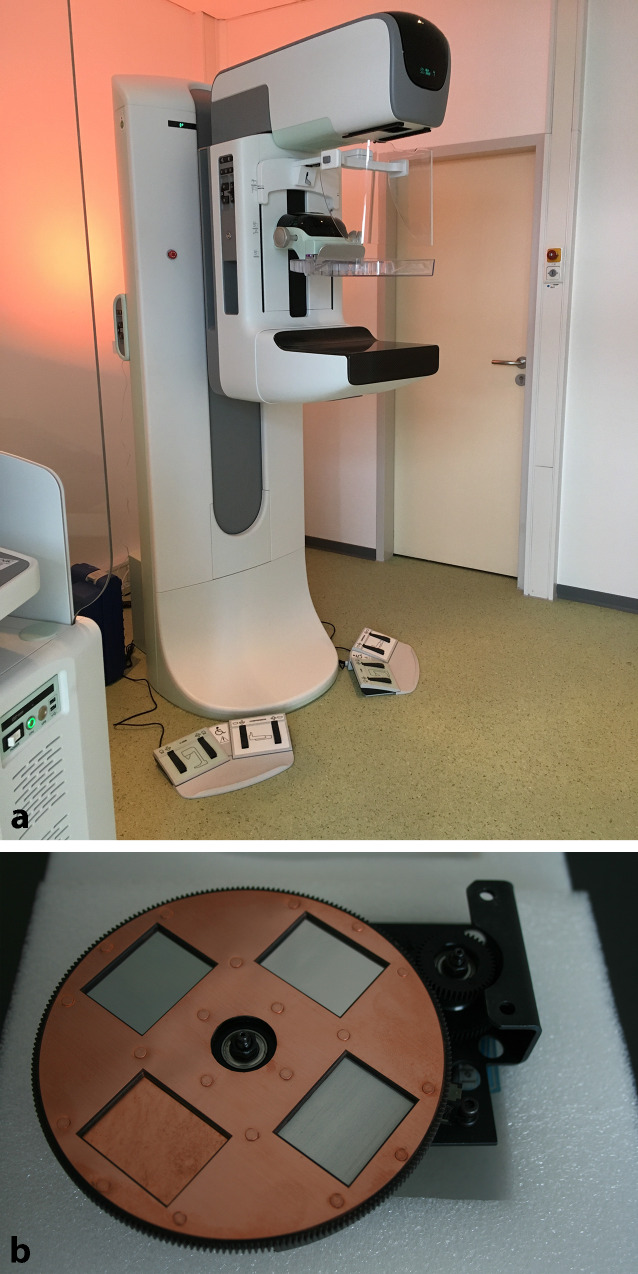

Die CEM basiert auf der Dual-Energie-Technologie. Das iKM wird intravenös appliziert und anschließend werden zwei Aufnahmen mit unterschiedlichem Röntgen-Energiespektrum erstellt, oberhalb (High-Energy) bzw. unterhalb (Low-Energy) der K‑Kante (33,2 keV) von Iod. Moderne Mammographie-Systeme (Abb. 1) ermöglichen die konsekutive Anfertigung je einer Low-Energy- (26–31 kV) und High-Energy-Aufnahme (45–49 kV) in derselben Kompression der Brust innerhalb weniger Sekunden.

**Figure 2:**
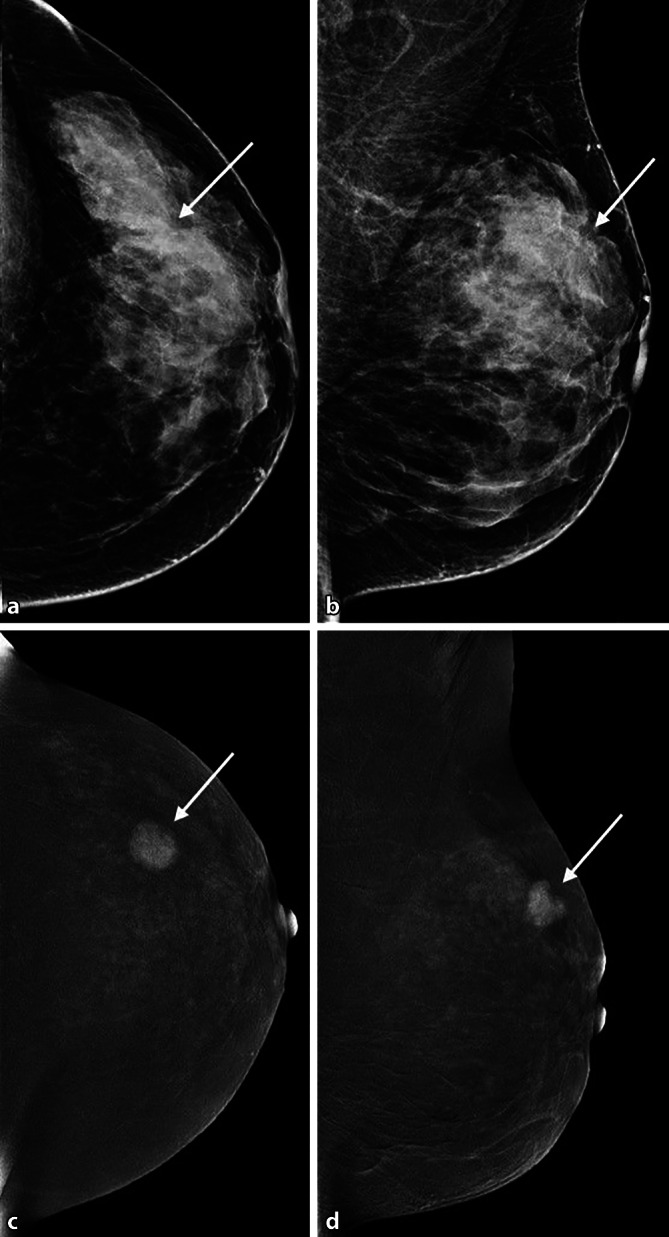

**Figure 3:**
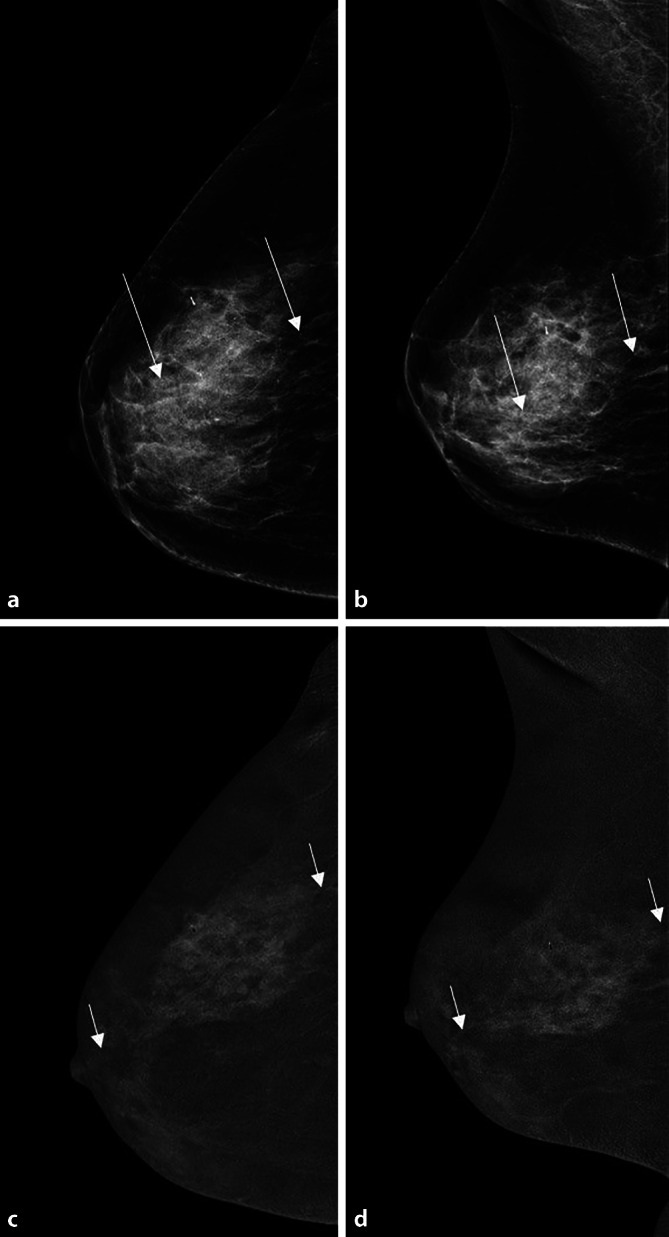

Die Low-Energy-Aufnahme weist den bekannten Bildcharakter der herkömmlichen Mammographie auf, die High-Energy-Aufnahme dient zur Darstellung der KM-Aufnahme und zur Berechnung des rekombinierten Bildes [26]. Basierend auf einem definierten Algorithmus wird das rekombinierte Bild generiert, in dem nur intramammäre Bereiche mit gesteigerter Iodanflutung hell kodiert sind (Abb. 2, 3, 4 und 5). Sind keine Bereiche hell kodiert, so ist dies ein Zeichen für eine fehlende umschriebene Kontrastmittelanreicherung in der Brust.

**Figure 4:**
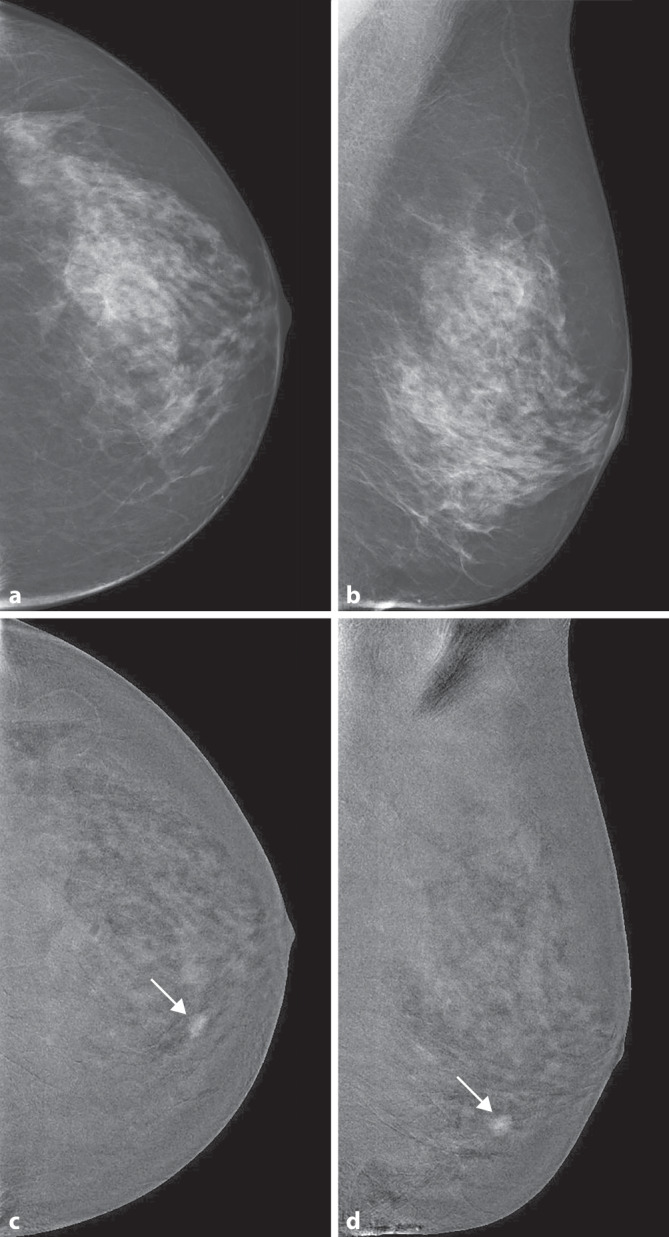

**Figure 5:**
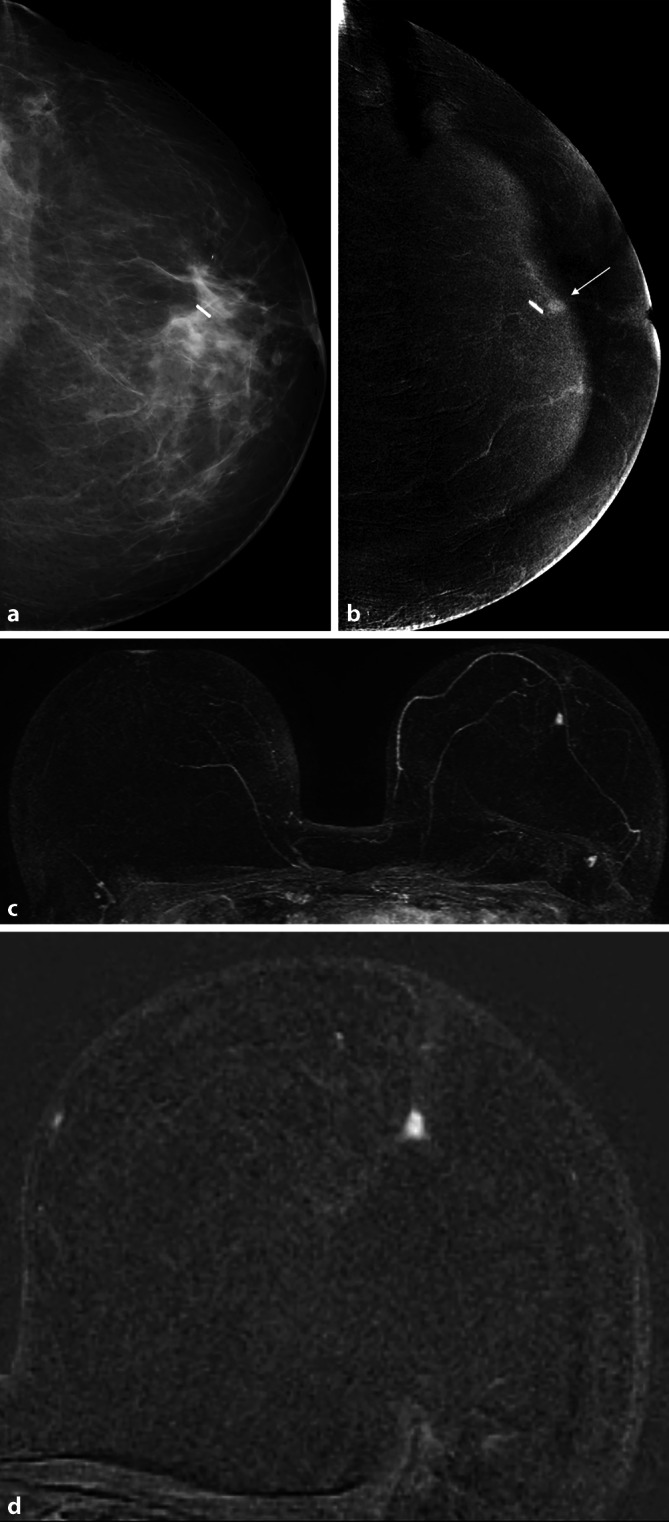

Die Algorithmen der unterschiedlichen Hersteller sind nicht identisch, sodass Unterschiede beim Bildeindruck der Low-Energy-Aufnahmen, des rekombinierten Bilds und der Artefaktausprägung auftreten können [25]. Eine gute Qualitätssicherung insbesondere bzgl. der Hintergrundsubtraktion muss sichergestellt werden.

## Methodik der CEM

Hinsichtlich der Methodik der CEM besteht mit Blick auf die Mitteilungen in der Literatur und die eigenen Erfahrungen noch einige Variabilität [7, 13, 19, 22, 28]. Weiterhin ungeklärte Aspekte der CEM betreffen z. B. die Reihenfolge der durchgeführten Untersuchungen nach KM-Gabe und deren Dokumentation, Dosis und Flow des Kontrastmittels sowie das Delay zwischen KM-Gabe und Anfertigung der Mammographien.

Die nachfolgenden Angaben sollen hier zumindest eine Orientierung geben: Die Autoren setzen vor allem iKM-Volumina von 100–150 ml mit 300–400 mg I/ml ein. Die Applikation erfolgt üblicherweise maschinell mit Flussraten von 3 ml/s [28]. Die Nachinjektion eines Bolus von mindestens 20 ml physiologischer Kochsalzlösung wird empfohlen [16]. Die Aufnahmen sollten innerhalb von 5 min –frühestens jedoch 2 min nach Injektionsstart beginnend – akquiriert werden. Standardaufnahmen werden beidseits in mediolateral-obliquer (MLO) und kraniokaudaler (CC) Projektion angefertigt. Im diagnostischen Setting ist es hierbei ratsam, mit der betroffenen Brust zu beginnen.

Die Mitteilung einzelner Arbeitsgruppen über eine gewichtsadaptierte iKM-Dosis von 1,5 ml/kg Körpergewicht ist eher kritisch zu sehen, da es sich bei der CEM um ein 2D-Verfahren handelt und daher Kontrastmitteldosierungen aus der 3D-Technologie nicht unbedingt übernommen werden können [28]. Hinzu kommt der Aspekt, dass es sich bei der CEM wie auch der Mamma-MRT um ein First-pass-Verfahren handelt, so dass die Anflutung des Kontrastmittels in der Brust im Wesentlichen vom Körpergewicht unabhängig ist.

## Parenchymdosis

Verfügbare CEM-Geräte erfüllen die Dosisgrenzwerte in der Mammographie mit einer durchschnittlichen mittleren Parenchymdosis von < 2,5 mGy/Aufnahme. Angemerkt sei, dass es sich hierbei um einen Durchschnittswert handelt. In Abhängigkeit von der individuellen Brustdrüsendichte findet sich eine Bandbreite von etwa 1,0 bis 5,0 mGy/Aufnahme. Da der Indikationsbereich der CEM in erster Linie Frauen mit hoher Gewebedichte betrifft, ist für diese Gruppe eher von einer höheren mittleren Parenchymdosis auszugehen.

Die Gesamtröntgendosis der CEM setzt sich zusammen aus der im Vergleich zur herkömmlichen Aufnahme identischen Low-Energy-Dosis und der zusätzlichen High-Energy-Dosis, die aufgrund der hohen Spannung nur etwa ein Viertel der herkömmlichen Aufnahmedosis beträgt. Im Rahmen der Abklärungsdiagnostik erscheint die Erhöhung der Parenchymdosis in der genannten Größenordnung eher akzeptabel. In einer Screeningkonstellation und hiermit verbundenen hohen Untersuchungszahlen gesunder Frauen ist das aus einer solchen Erhöhung der Gesamtdosis resultierende Lebenszeitrisiko allerdings anders zu bewerten [14]. Insgesamt ist die CEM-Strahlendosis geringer als bei einer Mammographie plus digitaler Brusttomosynthese (DBT).

Mit Blick auf den Aspekt der Parenchymdosis kann eine Überlegung sein, auf die zweite Aufnahmeebene (CC-Projektion) zu verzichten, da nicht oder gering anreicherndes Drüsengewebe bei der CEM nicht zu einer störenden Superposition eines KM-anreichernden Tumors führt. Bei Verzicht auf die CC-Projektion wäre in der Konsequenz sogar eine Reduktion der Parenchymdosis pro Brust um 30–40 % im Vergleich zur herkömmlichen 2‑Ebenen-Mammographie denkbar. Dies könnte Gegenstand zukünftiger wissenschaftlicher Evaluationen sein.

## Indikationen

Prinzipiell zeigen die KM-verstärkten Untersuchungsverfahren, u. a. KM-MRT und CEM, Vorteile gegenüber Mammographie und DBT bei der Detektion des Mammakarzinoms insbesondere bei Brustdichtetyp ACR C und D [9], da sie neben morphologischen Kriterien auch die Visualisierung der Angiogenese von Tumoren ermöglichen. So kommen für die CEM vordergründig alle Indikationen in Betracht, die auch für die KM-MRT der Brust als sinnvoll erachtet werden. In Abhängigkeit von den lokalen Gegebenheiten unterscheiden sich allerdings – mit Blick auf die Vergleichsdaten beider Verfahren verständlich – die Vorstellungen zu sinnhaften Indikationen der CEM. Diese *Spaltung* in zwei Lager findet sich auch innerhalb der Autorenschaft wieder:

Für Institute und Praxen, in denen neben der Mammographie, ggf. Tomosynthese und Ultraschall, keine Option zur Durchführung einer qualitätsgesicherten Mamma-MRT besteht, erscheinen alle Indikationen für eine CEM sinnvoll, die die Limitationen der vorhandenen diagnostischen Methoden überwinden.

Folgende Indikationen können in einer solchen Konstellation *ohne* MRT für die CEM diskutiert werden:

- Befundausdehnung und lokales Staging (Befundkategorie BI-RADS 4, 5 oder 6 und Dichtetyp ACR C oder D),
- Monitoring bei BI-RADS-6-Befunden unter neoadjuvanter Chemotherapie,
- postoperative Nachsorge nach brusterhaltender Therapie (BET) eines Mammakarzinoms,
- Primärtumorsuche bei unbekanntem Primarius (CUP-Syndrom),
- finale Abklärung unklarer Befunde in der Mammographie, DBT und Sonographie im kurativen Setting.

Für Institute und Praxen, in denen die Option zur Durchführung einer qualitätsgesicherten Mamma-MRT besteht, erscheint die CEM für deutlich weniger Indikationen sinnvoll:

- Vorliegen einer Kontraindikation für die KM-MRT bei gegebener Indikation zur MRT, z. B. Herzschrittmacher,
- Ablehnung durch die Patientin (z. B. Klaustrophobie, Kostenaspekte),
- Gadolinium-Unverträglichkeit,
- Adipositas per magna,
- frühzeitiges Assessment unklarer Befunde bei fehlender Option eines zeitnahen MRT-Termins.

Generell erscheint der Einsatz der CEM zum gegenwärtigen Zeitpunkt im Rahmen eines populationsbezogenen Mammographie-Screenings nicht indiziert, da hier noch keine ausreichenden Daten vorliegen. Diese Einschätzung kann sich nach Auswertung derzeit laufender prospektiver CEM-Screeningstudien in Amerika und Europa allerdings ändern. In diesem Zusammenhang werden insbesondere Aspekte der iKM-Gabe und der resultierenden Parenchymdosis erneut zu bewerten sein.

Der Einsatz der CEM im Brustkrebs-Screening bei Frauen mit definiertem Hochrisikoprofil verbietet sich ohnehin aufgrund der erhöhten Vulnerabilität dieser Gruppe gegenüber ionisierender Strahlung.

Letztendlich wird die Wahl der bildgebenden Methode neben der ärztlichen Empfehlung und der Kostenübernahme durch die Krankenkasse auch die individuelle Präferenz der zu untersuchenden Klientin bzw. Patientin mitentscheiden [12].

## Befundung

Die Anwendung der BI-RADS®-Deskriptoren aus der KM-MRT für die morphologische Analyse der CEM hat sich als sehr hilfreich erwiesen [17]. Im Jahr 2022 wurde die CEM in das ACR-BI-RADS®-Lexikon aufgenommen, ein wichtiger Schritt hin zu einer einheitlichen Befundung [2].

## KM-MRT der Brust vs. CEM

Eine Metaanalyse, die Studiendaten aus 2003 bis 2019 berücksichtigt und eine Gesamtanzahl von 945 Läsionen umfasst, belegt den Stellenwert der CEM. Danach erreicht die CEM eine als suboptimal bewertete Sensitivität von 85 % (95 % Konfidenzintervall [KI] 73–93 %) und eine Spezifität von 77 % (95 % KI 60–88 %) [23].

Bezüglich des direkten Vergleichs der CEM mit der Mamma-MRT zeigt eine andere aktuelle Metaanalyse den wohl zuverlässigsten Stand zum gegenwärtigen Zeitpunkt. Erfasst wurden 7 Studien respektive 1137 Läsionen (654 bösartig, 483 gutartig). Die Sensitivität der CEM zeigte sich hierbei genauso wie der negative prädiktive Wert (NPV) der MRT signifikant und klinisch relevant unterlegen. Die Spezifität lag in der CEM (74 %) gering höher als in der MRT (69 %) [21].

Einschränkend sei darauf hingewiesen, dass bisherige Studien bis auf einzelne Ausnahmen in erster Linie Indexläsionen bewerten, darunter auch konventionell detektierbare Befunde. Vergleichsstudien für die Detektion subtiler Veränderungen, z. B. im Rahmen des lokalen Stagings vor adäquater Therapie (extensives intraduktales Karzinom [EIC], Multifokalität, Multizentrizität) oder der Differenzierung von Low- und High-grade-DCIS (ductales Carcinoma in situ) liegen nicht vor. Erwähnt werden sollte auch, dass bei der CEM die Genauigkeit von Low-Energy-Aufnahme und Algorithmusbild kombiniert betrachtet wird. Die Selektion von mammographisch suspekten Befunden erhöht also die Sensitivität der CEM schon beim Auswahlkriterium.

Generell muss angemerkt werden, dass bei einzelnen Vergleichsstudien zwischen KM-MRT der Brust und CEM eine inadäquate Bildqualität in der MRT vorliegt, die bei weitem nicht den derzeit gültigen Standards einer qualitativ hochwertigen Mamma-MRT entspricht [27]. Solche Studien sind für die vergleichende Bestimmung des Stellenwerts der verschiedenen Untersuchungsverfahren nicht geeignet.

Anders als die CEM kommt die KM-MRT ohne ionisierende Strahlung aus, die insbesondere für jüngere Frauen relevant ist. Dies betrifft in besonderem Maße Frauen mit einem Gendefekt (z. B. BRCA), bei denen eine erhöhte Vulnerabilität der Brustzellen gegenüber ionisierender Strahlung besteht. Zudem erlaubt die multiparametrische KM-MRT eine höhere Gewebedifferenzierung und -charakterisierung. Aus Sicht der Autoren gibt es einige Punkte, die für die CEM sprechen, u. a. die größere Verfügbarkeit und ein höherer Untersuchungskomfort [12]. Außerdem sind häufig Mammographie-Voraufnahmen vorhanden, mit denen CEM-Aufnahmen verglichen werden können. In der kurativen Mammographie kann die CEM die Notwendigkeit zusätzlicher Mammographie-Aufnahmen ersetzen [8]. In der Low-Energy-Aufnahme werden intramammäre Kalzifikationen in gleicher diagnostischer Qualität wie bei der digitalen Vollfeld-Mammographie dargestellt [8]. Zudem sind die meisten medizinischen Technolog:innen in der Radiologie in der Anfertigung von Mammographie-Aufnahmen gut geschult. Die Entscheidung, ob die KM-MRT oder CEM zum Einsatz kommt, wird beeinflusst durch die Verfügbarkeit der jeweiligen Technologie, die Expertise des Anwendenden, die zu berücksichtigenden Kontraindikationen und durch den Wunsch der gut informierten Klientin bzw. Patientin.

## Abklärung von Befunden der Kategorie BI-RADS 4/5 und Befundmarkierung

Finden sich in der CEM-Befunde der Kategorie BI-RADS 4 oder 5, so sollte leitliniengemäß eine Sicherung des Befunds erfolgen, idealerweise minimal-invasiv mittels perkutan-bioptischer Abklärung. Wie auch bei auffälligen Befunden in der MRT sollte zunächst eine Second-look-Sonographie durchgeführt werden. Für die Biopsie und Clip- oder Drahtmarkierung von nur in der CEM detektierbaren Befunden stehen prinzipiell stereotaktische Interventionsvorrichtungen unter Einsatz der CEM zur Verfügung [1]. Die Befunde können hierbei nach der Anfertigung von Stereoaufnahmen, auf der Basis von Schrägaufnahmen von +15 und −15°, angesteuert werden. Hierbei ist ein Intervall von bis zu 20 min nach der Kontrastmittelgabe möglich, um die Zielläsion zu reproduzieren. Die mediane Interventionszeit in einer monozentrischen Studie zu CEM-gesteuerten Biopsien betrug 15 min [1]. Hierzu wird kritisch angemerkt, dass deren Anschaffung mit einer weiteren, nicht unerheblichen Kosteninvestition verbunden ist. Befunde der Kategorie BIRADS 4 oder 5 könnten alternativ MRT-gesteuert biopsiert oder markiert werden, sofern nicht ohnehin primär eine Kontraindikation für die MRT zum Einsatz der CEM geführt hat. Letztendlich stellt die notwendige perkutane bioptische Abklärung von Befunden der Kategorie BIRADS 4 oder 5 in der CEM weiterhin ein weitestgehend ungelöstes Problem dar. Spezielle CEM-gesteuerte Interventionssysteme machen in diesem Zusammenhang sicherlich nur Sinn, wenn entsprechende Untersuchungszahlen vorliegen.

## Diskussion

Mit der CEM steht neben der Mamma-MRT eine weitere Methode zur Verfügung, mit der sich die Angiogenese maligner Tumoren in der Brust visualisieren lässt. Sie ist damit allen anderen bildgebenden Untersuchungsverfahren, die ohne Kontrastmittel arbeiten, insbesondere bei Frauen mit dichten oder sehr dichten Brüsten im Nachweis von Brustkrebs deutlich überlegen. Die CEM zeichnet sich durch Aufnahmezeiten im Bereich von 10 min aus und liegt damit in einer Größenordnung moderner MRT-Konzepte. Zudem ist diese Methode in verschiedenen Szenarien sofort verfügbar. Eine prinzipielle Ausweitung der Verfügbarkeit der CEM könnte sich mit vergleichsweise geringem Ressourcenaufwand erreichen lassen. Wie bei der herkömmlichen Röntgenmammographie kommt auch bei der CEM naturgemäß ionisierende Strahlung zu Einsatz. Damit sollte die CEM bei jungen Frauen und insbesondere bei Frauen mit erhöhtem genetischem Risiko für das Mammakarzinom nicht durchgeführt werden.

Eine weitere relative Limitation betrifft die Notwendigkeit der intravenösen KM-Applikation. Dies gilt insbesondere für den Einsatz der CEM im Rahmen von Screeningkonzepten, da hier asymptomatische gesunde Frauen untersucht werden und die Risikoabwägung bezüglich eines potenziellen Kontrastmittelzwischenfalls anders ausfällt als in der klinischen Routine. Schlussendlich fehlt für den Einsatz der CEM im Screening derzeit noch die Evidenz, anders als beim Screening-MRT, für das die DENSE-Studie den Nutzen gegenüber der Mammographie bei Frauen mit extrem dichtem Brustgewebe aufzeigen konnte [3]. Zu dieser Thematik werden in Kürze Ergebnisse und aktualisierte Bewertungen laufender CEM-Studien in Europa und USA erwartet. In jedem Fall müsste in einer Screening-Einheit die ärztliche Erreichbarkeit *einheitsnah* sichergestellt werden. Unabhängig von der KM-Gabe ist die um etwa 20 % höhere Strahlendosis bei Einsatz der CEM im Rahmen eines Brustkrebs-Screenings kritisch zu sehen. In diesem Zusammenhang ist die Reduktion auf eine 1‑Ebenen-CEM denkbar, die im Vergleich zur etablierten 2‑Ebenen-Mammographie eine Dosisreduktion mit sich bringen würde. Hierzu gibt es allerdings (noch) keine Daten.

Metaanalysen zum Vergleich der CEM mit der KM-MRT belegen eine signifikant höhere Sensitivität und NPV der MRT. Bezüglich der Spezifität zeigt die CEM gering bessere Daten als die MRT. Anzumerken ist, dass es noch keine aussagekräftigen Vergleichsstudien zur Detektion von sehr kleinen Brusttumoren gibt. Hier zeigen die modernen Protokolle der qualitätsgesicherten KM-MRT mit Untersuchungszeiten von maximal 10 min und einer Durchschnittsgröße der detektierten Karzinome um 8 mm sehr viel Potenzial [5, 10–12, 18].

Das Indikationsspektrum zur CEM kann gegenwärtig wohl am ehesten in Abhängigkeit von den lokalen Gegebenheiten gesehen werden. Für Institute und Praxen, in denen keine qualitätsgesicherte Mamma-MRT zur Verfügung steht, erscheinen alle Indikationen für eine CEM sinnvoll, die die Limitationen von Mammographie, DBT und Ultraschall überwinden. Für Institute und Praxen mit qualitätsgesicherter Mamma-MRT reduzieren sich die Indikationen auf Konstellationen, in denen MRT nicht oder nicht zeitnah möglich ist.

## Fazit für die Praxis

- Kontrastmittelverstärkte Untersuchungsverfahren wie die Magnetresonanztomographie (MRT) mit Kontrastmittel (KM) und die kontrastmittelverstärkte Mammographie (CEM) erweisen sich in der bildgebenden Mammadiagnostik gegenüber der Mammographie hinsichtlich Sensitivität und Spezifität überlegen, insbesondere bei Frauen mit hoher Brustdichte.
- Bezüglich der Methodik der CEM besteht noch Variabilität.
- Das Indikationsspektrum zur CEM kann gegenwärtig in Abhängigkeit von den lokalen Gegebenheiten gesehen werden. Steht eine qualitätsgesicherte Mamma-MRT zur Verfügung, erscheinen Indikationen für eine CEM sinnvoll, welche die Limitationen von Mammographie, Brusttomosynthese (DBT) und Ultraschall überwinden.
- Mit der Option einer qualitätsgesicherte Mamma-MRT reduzieren sich die Indikationen für eine CEM deutlich.
- Das iodhaltige Kontrastmittel Iopromid (Ultravist®) hat im Januar 2023 auf EU-Ebene die Indikationserweiterung für die CEM erhalten.
- Neben der ärztlichen Empfehlung und der Kostenübernahme durch die Krankenkasse wird letztendlich auch die individuelle Präferenz der zu untersuchenden Frau die Wahl der bildgebenden Methode beeinflussen.
